# Field and laboratory microplastics uptake by a freshwater shrimp

**DOI:** 10.1002/ece3.11198

**Published:** 2024-04-01

**Authors:** Ross N. Cuthbert, Masimini S. Nkosi, Tatenda Dalu

**Affiliations:** ^1^ Institute for Global Food Security, School of Biological Sciences Queen's University Belfast Belfast UK; ^2^ Aquatic Systems Research Group, School of Biology and Environmental Sciences University of Mpumalanga Nelspruit South Africa

**Keywords:** aquatic ecosystem, *Caridina nilotica*, functional response, maximum feeding rate, pollution, South Africa

## Abstract

Microplastics are widespread pollutants, but few studies have linked field prevalence in organisms to laboratory uptakes. Aquatic filter feeders may be particularly susceptible to microplastic uptake, with the potential for trophic transfer to higher levels, including humans. Here, we surveyed microplastics from a model freshwater shrimp, common caraidina (*Caridina nilotica*) inhabiting the Crocodile River in South Africa to better understand microplastic uptake rates per individual. We then use functional response analysis (feeding rate as a function of resource density) to quantify uptake rates by shrimps in the laboratory. We found that microplastics were widespread in *C. nilotica*, with no significant differences in microplastic abundances among sampled sites under varying land uses, with an average abundance of 6.2 particles per individual. The vast majority of microplastics found was fibres (86.1%). Shrimp microplastic accumulation patterns were slightly higher in the laboratory than the field, where shrimp exhibited a hyperbolic Type II functional response model under varying exposure concentrations. Maximum feeding rates of 20 particles were found over a 6 h feeding period, and uptake evidenced at even the lowest laboratory concentrations (~10 particles per mL). These results highlight that microplastic uptake is widespread in field populations and partly density dependent, with field concentrations corroborating uptake rates recorded in the laboratory. Further research is required to elucidate trophic transfer from these taxa and to understand potential physiological impacts.

## Graphical abstract

Microplastics are widespread pollutants, but few studies have linked field prevalence in organisms to laboratory uptakes. Our results highlight that microplastic uptake is widespread in field populations of shrimp and partly density dependent, with field concentrations corroborating uptake rates recorded in the laboratory.

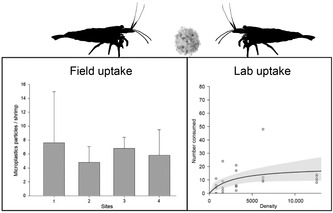

## 1 INTRODUCTION

Microplastics (<5 mm) are ubiquitous and persistent anthropogenic pollutants (GESAMP, 2019). They are intentionally manufactured for personal care products (primary microplastics) or are broken down via the fragmentation of larger plastics (secondary microplastics; Reynolds & Ryan, 2018). Most inland water microplastics are released from industrial areas as untreated and treated wastewater (Nan et al., 2020; Windsor et al., 2019), eventually reaching marine environments (Dalu et al., 2019). Microplastics contain chemical additives and have a large surface–to–volume ratio that allows the absorption of dissolved chemicals, which may additionally threaten the health of aquatic organisms (Nel et al., 2018).

Recent studies have highlighted that aquatic organisms can actively ingest microplastics or indirectly ingest them through trophic transfer at different levels [e.g., by crustaceans (Peixoto et al., 2019), bivalves (van Cauwenberghe & Janssen, 2014), and fish (Zakeri et al., 2020)]. In addition to ingestion and trophic transfer, other microplastics uptake routes are possible, such as via gill chambers through respiration (Gray & Weinstein, 2017). The possible effects of transfer activities include reduced fecundity (Troost et al., 2018) and lower food intake and growth (Foley et al., 2018).

Similar to other aquatic organisms, herbivorous–feeding organisms are highly susceptible to microplastics ingestion (Rahman, 2019), due to their feeding behaviours which involve filtering small items suspended in the water column. Hence, they often cannot avoid ingesting microplastics materials similar to their preferred food size (Cole et al., 2013). Shrimps are considered important biological indicators of microplastics pollution (Gonçalves et al., 2019), and studies have highlighted that shrimps can effectively ingest microplastics in the field (Nan et al., 2020; Tongnunui et al., 2022) and laboratory (Gray & Weinstein, 2017; Wang et al., 2021). They are also generally available to large freshwater predators, such as crabs, fish, and birds (Crowl et al., 2001). Predators such as fish are usually a source of protein to humans, and microplastics can accumulate in fish tissue such as the gut, gills, liver, and brain (Ding et al., 2019), which then poses a risk to human health (Prata et al., 2020; Vethaak & Legler, 2021). This highlights the potential contribution of shrimp in the trophic transfer of microplastics across freshwater systems and necessitates studying microplastics uptake in aquatic and into the terrestrial environments.

The atyid shrimps of the genus *Caridina* are mostly found in riverine habitats and are small taxa which carry a few eggs, playing an ecological role as herbivorous‐detritivorous feeders (Hart, 1981). The taxa are obligatorily fluvial, widely‐distributed, and abundant. *Caridina nilotica* is extensively distributed in riverine and standing waters of southern Africa, mostly feeding on debris and epiphytic microflora, and they also form a major dietary component of most fishes (Richard & Clark, 2005). For example, in Lake Sibaya, *C. nilotica* was observed to breed perennially, with high egg stocks and instantaneous birth rates in summer. Females were generally larger than males within this system, with clutch size increasing linearly with female carapace length (Hart, 1981). Another shrimp species *Atya innocous* collects food by filtering particulate matter from the water column and by sweeping or scraping the substrate, similar to *Caridina* spp. (Felgenhauer & Abele, 1983).

Microplastic uptake by organisms can differ depending on the microplastics density present in the environment (Drago et al., 2020). Nevertheless, there is a general lack of knowledge as to how the organism's microplastics uptake rate responds to varying microplastics concentrations (Mbedzi et al., 2020). In many applied and fundamental ecological fields, functional responses (FRs) examine the relationship between consumption rate and resource density (e.g., Cuthbert et al., 2019; Holling, 1959). This study uses FRs to measure resource (MPs) utilisation as a function of resource density (MPs dosage). There are three forms of FRs (Holling, 1959), where: (i) Type I is characterised by a linear relationship between consumption rate and resource density, until the rate is saturated, (ii) Type II exhibits a curvilinear increase where the resource ingestion rate decreases asymptotically with increasing resource density, and (iii) Type III, which is associated with a sigmoid–shaped response, where the resource ingestion rate is low when the resource concentration is low and then rises before reaching an asymptote (Drago et al., 2020; Jeschke et al., 2004). However, despite FRs being a widely used approach in ecology, there has been only a few examples of its application in microplastic uptake quantification (Drago et al., 2020; Mbedzi et al., 2020; Woods et al., 2018).

Thus, the present study aimed to (i) survey field uptake by a model freshwater shrimp *Caridina nilotica* and to (ii) employ FRs to measure the experimental uptake of microplastics by this shrimp species at different densities. This will inform the level of pollution typically encountered in this species, and the propensity for uptake in the laboratory. Shrimp were sampled from the Crocodile River in South Africa. The Crocodile River is an essential water source for agriculture, with a 10,440 km^2^ catchment area. Within the middle reaches is the Mpumalanga Province capital Nelspruit, which is home to most industrial activities, wastewater treatment plants, and storm drainage facilities, all of which are potential sources of microplastics (Eriksen et al., 2013). We hypothesise that shrimps will ingest plastics in all field sites and that laboratory uptake will be positively density dependent with concentrations. We also anticipate that laboratory uptake will be higher than in the field.

## 2 MATERIALS AND METHODS

### 2.1 Study area

**FIGURE 1:**
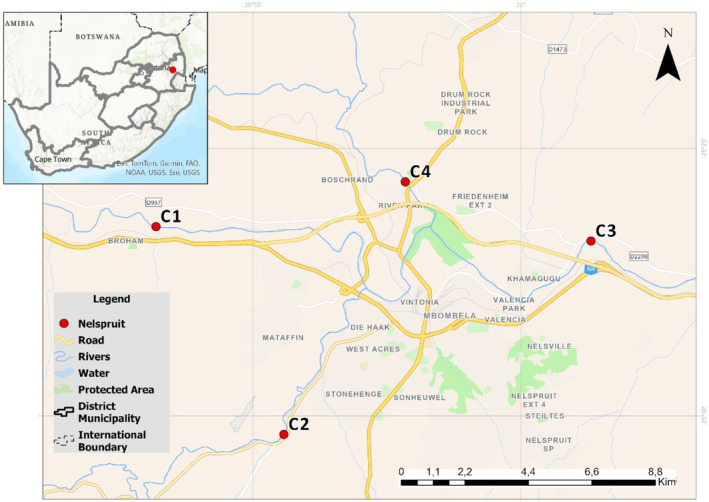
Location of the field study sites within the Nelspruit city area, South Africa.

Four sites were surveyed along the Crocodile River around the Nelspruit (Mbombela) town in May 2022 as previous surveys have indicated the presence of shrimp within them. Sites 1 (upstream of town; −25.441338, 30.887864) and 3 (downstream of town; −25.445179, 31.021767) were located along the mainstem of the Crocodile River. Site 2 was upstream of the Gladdespruit River (−25.506185, 30.925794), a tributary of the Crocodile River, which was located next to illegal domestic waste and rubble dump site. Site 4 was situated in Nel's River (−25.426909, 30.964544), a tributary to the Crocodile River located downstream of an Elawini estate, with frequent pumping of water for construction (Figure 1). The mean range of water temperature, pH, conductivity and total dissolved solids was 18.3–19.6°C, 7.4–7.9, 256.5–658.5 μS/cm, and 96.6–256.4 mg/L, respectively. The microplastic concentrations ranged from 250 to 2600 particles m^−3^ (mean: 1058 m^−3^) within the Crocodile River during the cool‐dry season (Nkosi et al., 2023).

### 2.2 Field collections

We used a handheld bottom kick net to sample for freshwater shrimp *C. nilotica* along a 10 m transect in the littoral zones of the Crocodile River system and its tributaries from each of the four sites. At each site, two samples of shrimps (each having five shrimps except for site 3, which had only five shrimps in total) were collected from different zones or areas of the river to obtain a representative sample and preserved immediately in 70% alcohol in labelled containers for laboratory examination.

For the laboratory FR experiment, about 60 additional *C. nilotica* shrimps were collected from site 2 in a similar manner. We sampled site 2 only for practical reasons. All sampled shrimp were placed in 3 × 15 L buckets half–filled with filtered (i.e., through 20 μm mesh) site water. The *C. nilotica* used within the experiments measured 2.4 ± 0.3 cm (± standard error) in length. A further 30 L of source water was collected for experimental media. The *C. nilotica* shrimps were transported to the University of Mpumalanga Laboratory, where they were placed into an 80 L glass tank with filtered (20 μm mesh size) river water, where the water was continuously aerated at room temperature 24°C under 12:12 h light:dark conditions.

### 2.3 Experimental design

Our experiment broadly followed the methodology reported by Mbedzi et al. (2020). Shrimp were starved for 24 h, so as to empty their guts from food and potential microplastics. Experimentation and acclimation were undertaken under a 12:12 light:dark laboratory photoperiod. The *C. nilotica* used within the experiments measured 2.4 ± 0.3 SE cm in length. After 24 h, individuals were placed in 80 mL (volume 160.1 cm^3^) of filtered river water (through a 2 μm sieve) within glass containers of 5.6 cm diameter to acclimatise for an additional 4 h. The *C. nilotica* were then randomly presented with six different densities of 125 μm particle size of surface–modified white polyethene powder (Sigma–Aldrich, UK) (0.5 g (784 particles; ~4.9 particles cm^−3^), 1 g (1568 particles; ~9.8 particles cm^−3^), 2 g (3136 particles; ~19.6 particles cm^−3^), 4 g (6272 particles; ~39.2 particles cm^−3^), 8 g (12,544 particles; ~78.4 particles cm^−3^), and 16 g (25,088 particles; ~156.7 particles cm^−3^)), with five replicates per density and a total of 35 individual *C. nilotica* shrimps, including the controls. The controls consisted of five replicates with no microplastics, to ensure microplastics uptake related solely to its intentional supply. The experimental treatments were randomised and simultaneously run for 6 h without any food provided. Shrimps were euthanised in 70% alcohol immediately after the experiment and were carefully placed in rinsed pre–labelled individual test tubes.

### 2.4 Microplastic quantification

To prevent contamination, all surfaces and equipment were cleaned with milliQ distilled water prior to laboratory analysis. Furthermore, the air‐conditioner was not utilised during microplastic extraction process to minimise the potential risk of air‐borne microplastic particle contamination. During the microplastic extraction process, all glassware used was covered with aluminium foil, and laboratory coats were worn all the time to prevent further contamination.

Approximately 20 mL of nitric acid (55%) was added to digest the field and laboratory shrimps (as whole individuals) for 2 h, followed by boiling the sample if any organic matter was still visible. The solution was then diluted with distilled water and filtered through 2 μm mesh pore mixed cellulose ester Whatman membrane filters (47 mm diameter) using a vacuum pump, before being placed in a labelled petri dish, and then allowed to dry at room temperature over 72 h. The microplastics were identified and quantified using a Carl Zeiss Stemi dissecting microscope (Carl Zeiss MicroImaging GmbH, Göttingen) at ×40 to ×200 magnification based on shape and morphometric types (i.e., fibres, fragments, beads, foam) for the field studies. All identified microplastics were verified using the Nile Red dye (CAS 7385‐67‐3, HYD0718–500 mg, Hycultec, Beutelsbach) as it exploits the hydrophobic properties of microplastic by staining them and illuminating it under a blue fluorescein light, despite its potential to stain biological samples. Once stained, a blue light fluorescein was used to fluoresce and identify the initially suspected microplastics (Nalbone et al., 2021; Nkosi et al., 2023). Microplastic sizes were quantified based on the stage micrometre and an eyepiece reticle after first initial calibrating and results were presented in micrometres (μm).

### 2.5 Data analysis

A Poisson generalised linear model was used to test for differences in shrimp microplastic abundances among sites across particle types using R v4.3.1. A likelihood ratio test was then used to report the main effect of site on overall microplastic abundance.

For the functional responses experiment. The numbers of microplastic particles consumed were analysed as a function of starting supply treatment levels using a generalised linear model assuming a quasi–Poisson distribution. This error family was used to account for residual overdispersion compared to degrees of freedom in the model.

Logistic regression was used to analyse proportional microplastics consumption as a function of initial microplastics abundance. From this regression, a significantly negative first–order term indicates a Type II functional response, whilst a significantly positive first–order term followed by a significantly negative second–order term indicates a Type III functional response (Juliano, 2001). Type I functional responses are linear with increasing density. The functional response was Type II and thus modelled using Rogers' random predator equation owing to the non–replacement of microplastics particles during the experiment (Rogers, 1972):

(1) $$N_{e}=N_{0}(1-\exp\left(a\left(N_{e}h-T)\right)\right)$$

where *N*_e_ is the number of microplastics consumed, *N*_0_ is the initial density of particles, *a* is the attack rate, *h* is the handling time, and *T* is the experiment duration (fixed at 1, i.e., 6 h). The Lambert W function was used to allow for model fitting, owing to the recursive nature of the random predator equation (Bolker, 2008). A non–parametric bootstrapping procedure (*n* = 2000) was followed to generate 95% confidence intervals (Cis) around the functional response curve (Pritchard et al., 2017).

## 3 RESULTS

**FIGURE 2:**
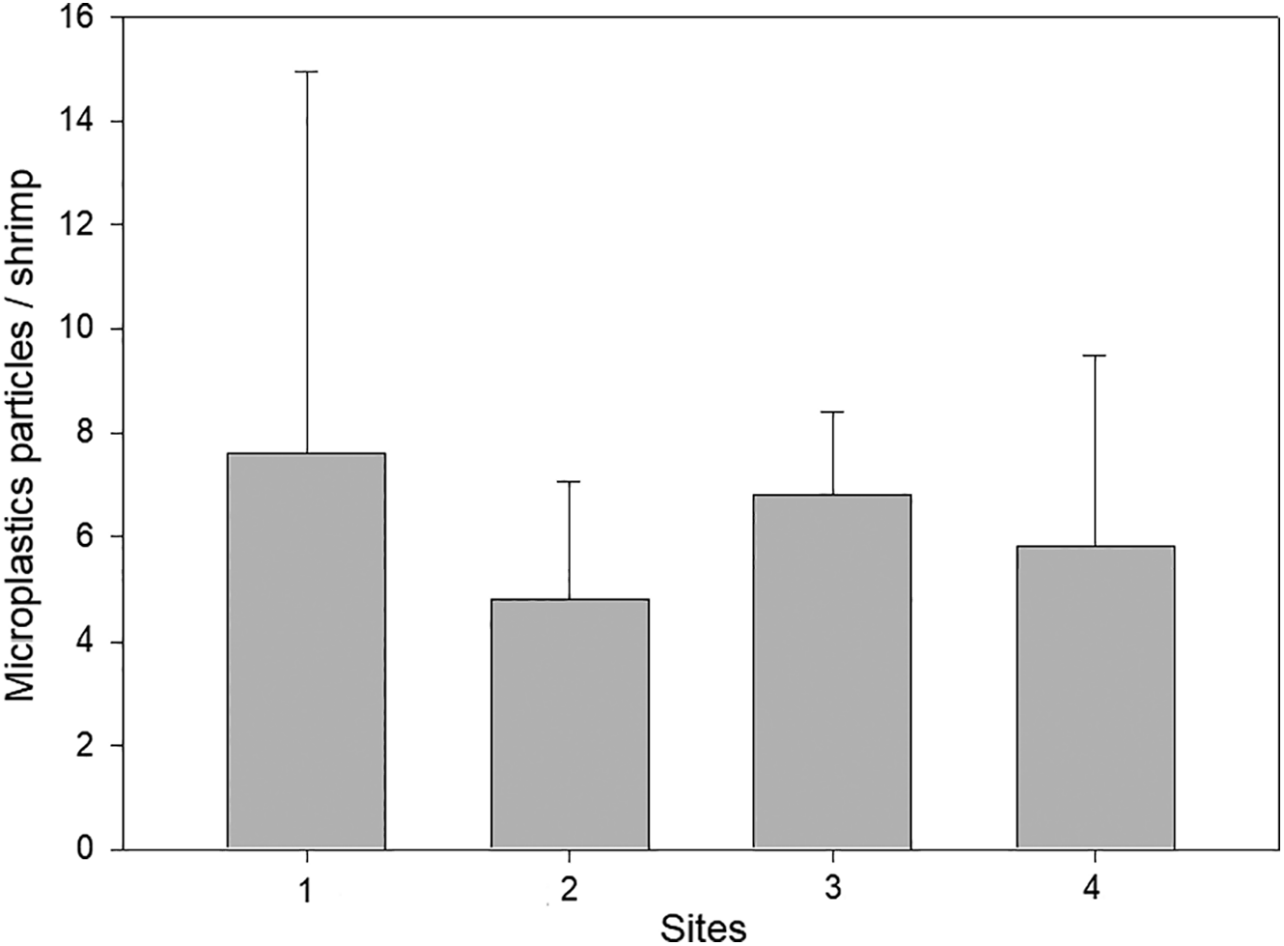
Mean microplastics uptake per freshwater shrimp *Caridina nilotica* in the Crocodile River system, South Africa. Means are shown with standard deviation.

Based on the *C*. *nilotica* shrimps collected from sites 1, 2, 3 and 4, a total of 216 microplastics particles (*n* = 35) was identified, with an average abundance of 6.2 microplastics particles per individual and a mean range from 4.8 to 7.6 across sites (Figure 2). The majority of microplastics found in *C. nilotica* guts were fibres (86.1%; size range 25–360 μm), with only fragments (5.1%; size range 25–150 μm), foam (3.7%; size range 25–100 μm), beads (3.2%; size range 25–200 μm), and film (1.9%; size range 100–200 μm) observed across the sites. Microplastics uptake by shrimps had no significant difference (GLM: Chi‐square = 1.156, df = 3, *p* = .282) among the study sites.

**FIGURE 3:**
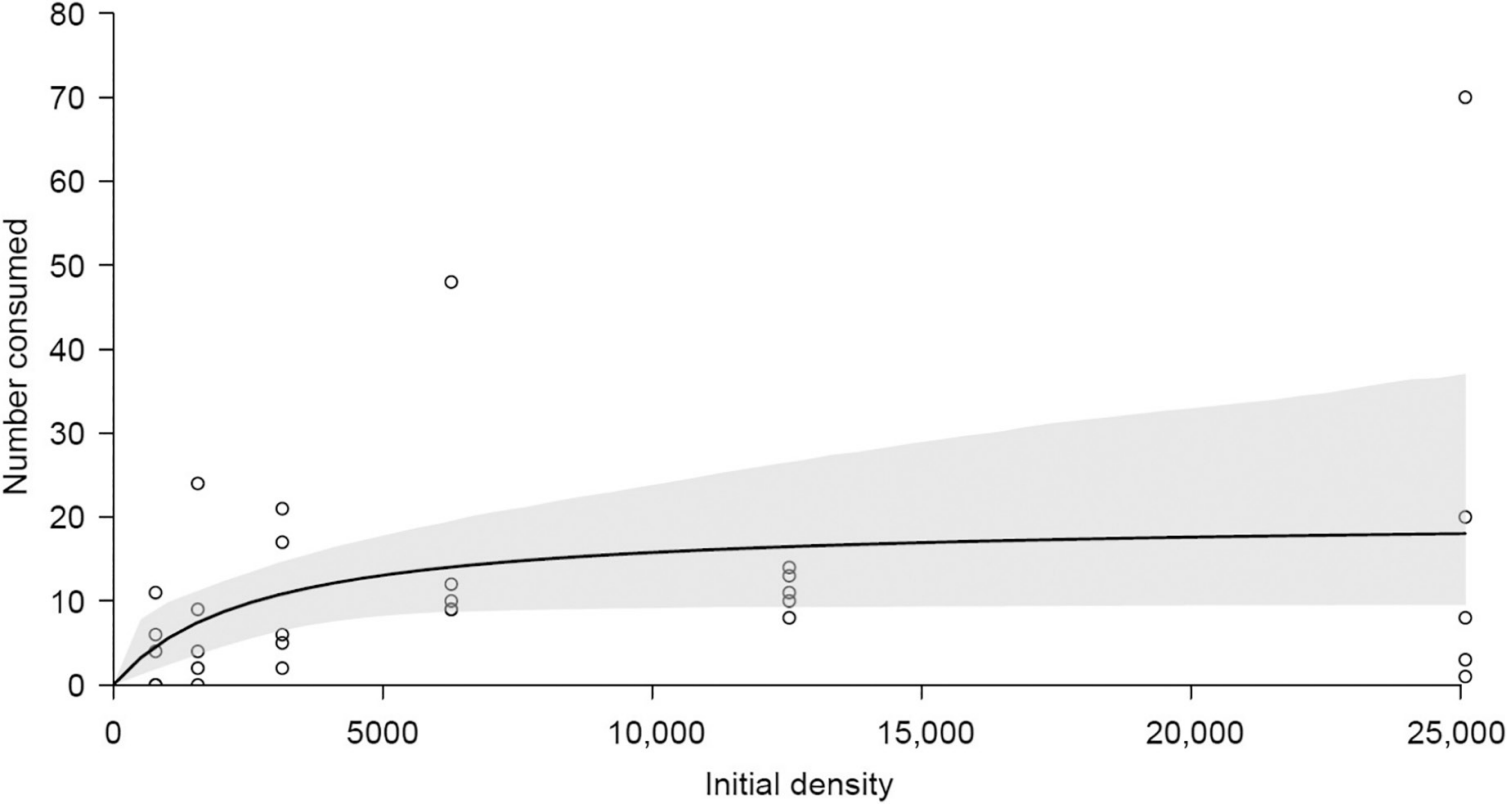
Type II functional response of *Caridina nilotica* consuming microplastics when exposed to different initial experimental microplastic densities. The *y*‐axis refers to numbers of microplastic particles consumed per shrimp. Shaded areas are bootstrapped 95% confidence intervals, and points are raw data. Functional response modelling was performed via Rogers' random predator equation.

In the FR experiment, no microplastics were consumed in particle–free control shrimps, and this group was therefore removed from further analyses. The lack of particles in controls confirms that the shrimp had cleared their guts and that there was no contamination. Microplastics were found in 90% of exposed shrimps, and counts tended to increase with greater exposure concentrations (Figure 3). Nevertheless, there were no statistically clear differences in microplastics consumption among exposure concentrations (quasi–Poisson GLM: *t* = 1.828, *p* = .078), with shrimps consistently consuming particles even when relatively sparse in the environment.

The proportion of microplastic consumed was significantly negatively related to the starting density (binomial GLM: *z* = 11.540, *p* < .001). Therefore, shrimps exhibited significant evidence for a hyperbolic Type II functional response (Figure 3). This enabled functional response attack rate (*a*) and handling time (*h*) parameter estimations to be returned (random predator equation: *a* = 0.008, *z* = 5.459, *p* < .001; *h* = 0.050, *z* = 10.697, *p* < .001). Accordingly, shrimps exhibited maximum consumption rates (1/*h*) of approximately 20 particles over the 6 h experimental period (Figure 3).

## 4 DISCUSSION

Microplastics were found in all field sampled freshwater shrimp and uptake was shown to be partly density dependent in the laboratory in the present study. While we hypothesised that uptake would be positively density dependent, we found that uptake displayed a hyperbolic relationship with exposure concentrations. In the field survey, microplastics accumulation in *C. nilotica* from the Crocodile River and its tributaries had a mean range of 4.8 to 7.6 particles per individual across sites, with an average of 6.2 particles per individual. This quantity is slightly lower than maximum feeding rates in the laboratory, supporting our second hypothesis and suggesting that microplastics may rapidly pass through the shrimp or reflect lower environmental concentrations encountered in the field. This abundance is greater than Nan et al. (2020), who found an average of 0.52 ± 0.55 piece/individual in shrimps of *Paratya Australianises* and Tongnunui et al. (2022), who found a similar average of 0.46 ± 1.64 piece/individual in shrimps of *Macrobrachium lanchesteri*. Most of the shapes of microplastics found in the study were fibrous (86.1%); interestingly, Nan et al. (2020) and Tongnunui et al. (2022), also observed similar trends. While the sampled sites here varied in terms of their characteristics, such as some being upstream of wastewater treatments, agricultural areas, rubble and waste dumping sites and water collection sites, there was no significant difference in uptake, suggesting widespread pollution in the Crocodile River. Further research is needed to elucidate the environmental drivers of microplastics concentrations among these sites.

Our experiment demonstrates that adult *C. nilotica* can ingest microplastics even at the lowest supplied experimental microplastics concentrations, likely owing to similarities in the shape and size with their preferred microalgal food. Uptake generally increased with microplastics exposure concentrations, but with no statistically clear difference among concentrations. Higher microplastics concentrations were found inside mysid shrimps *Neomysis integer* individuals in relatively high microplastics concentrations (10^3^–10^4^ particles mL^−1^) than in the present study (Setälä et al., 2014). Studies have widely reported associated adverse impacts on juvenile and adult shrimps, such as mortality in adult daggerblade grass shrimps (Gray & Weinstein, 2017); reduced microalga feeding in brine shrimp *Artemia parthenogenetic* larvae (Wang et al., 2021); and affected reproduction success in juvenile and adult shrimps of *Artemia franciscana* (Peixoto et al., 2019). The finding observed in the present study highlights that *C. nilotica* might be affected by microplastics' negative impacts even when their concentrations are low in the environments (Cunningham & Sigwart, 2019; Mbedzi et al., 2020), but further studies are needed to elucidate these potential impacts across concentrations. Further studies are also needed to uncover the effects of other polymer types on uptake rates. Here, our use of polyethylene could have reduced levels of ingestion due to its low density and propensity to float at the surface of waters, rather than in the zone occupied by the studied shrimp.

*Caridina nilotica* microplastics uptake demonstrated significant non–linear density dependence in the present study, with an asymptotical decrease in microplastics consumption rate with increasing environmental microplastics concentrations. Following the Type II FR, where the uptake rate is higher under low environmental microplastics concentration (Holling, 1959), this demonstrates that *C. nilotica* efficiently ingested microplastics even when relatively sparse in the environment. Drago et al. (2020) observed a similar response in freshwater rotifer *B. calyciflorus* across three microplastics sizes (i.e., 1, 3, 6 μm) and with different concentrations than in the present study. The attack rate corresponds to the ability of *C. nilotica* to detect and clear microplastics particles per unit time (i.e., curve initial slope), whilst the handling time relates to the capacity of *C. nilotica* to process microplastics particles in a given time unit. Handling time estimates inversely exhibited microplastics' maximum consumption rate (curve asymptote). Uptake of microplastics using functional response analyses has also been shown in a widespread African fish species *Tilapia sparmanii* (Mbedzi et al., 2020); in blue mussel *Mytilus edulis* (Woods et al., 2018); and in a common freshwater rotifer *Brachionus calyciflorus* (Drago et al., 2020).

## 5 CONCLUSIONS

The freshwater shrimp *C. nilotica* can efficiently consume microplastics even under relatively low densities and with consistent concentrations taken up in the field. However, our experiment did not include environmental conditions, such as turbidity and habitat structure which might alter aquatic organisms' response to microplastics, or measure environmental microplastics concentrations. Moreover, the field‐based uptake included a variety of microplastic forms (e.g., fibres) that were standardised in the laboratory experiment with the use of particles. Thus, in future, the response of aquatic organisms to different forms of microplastics should be observed across a gradient of observed environmental conditions to help understand microplastics' impact, and to relate laboratory exposure studies to naturally occurring concentrations. Future studies should additionally consider the physiological impacts of microplastics on these biota.

## AUTHOR CONTRIBUTIONS

**Ross N. Cuthbert:** Conceptualization (equal); formal analysis (equal); supervision (equal); visualization (equal); writing – original draft (equal). **Masimini S. Nkosi:** Formal analysis (equal); investigation (equal); writing – original draft (equal). **Tatenda Dalu:** Conceptualization (equal); formal analysis (equal); supervision (equal); visualization (equal); writing – original draft (equal).

## FUNDING INFORMATION

This study was funded by the National Research Foundation Thuthuka grant (#138206), and RNC was funded by the Leverhulme Trust (#ECF‐2021‐001).

## CONSENT TO PARTICIPATE

All authors consented to the participation in this manuscript and were fully involved.

## Supporting information


Data S1

## Data Availability

Underlying data will be made available in the Supplementary Material S1.
