# Supplementary material for: Field and laboratory microplastics uptake by a freshwater shrimp
Source: Ecol Evol. 2024 Apr 1;14(4):e11198. doi: 10.1002/ece3.11198 (PMC10985367; doi:10.1002/ece3.11198)
Supplement: Supplementary file 1 — Data S1 [file ECE3-14-e11198-s001.zip › legends.docx]

Caption: Supplementary data for the microplastics analyses in this study.
